# Effectiveness of Proton Pump Inhibitor Therapy in the Prevention of Bleeding After Prophylactic Endoscopic Variceal Band Ligation

**DOI:** 10.7759/cureus.33932

**Published:** 2023-01-18

**Authors:** Imran Khawaja, Muhammad Babar, Shakeel Ahmad Awan, Asif J Shaikh, Adnan A Abbasi

**Affiliations:** 1 Internal Medicine, Ayub Teaching Hospital, Abbottabad, PAK; 2 Medicine, University Hospitals Birmingham NHS Foundation Trust, Birmingham, GBR; 3 Medicine, University Hospitals of Derby and Burton, Burton, GBR; 4 Nephrology, Zayed Military Hospital, Abu Dhabi, ARE; 5 Family Medicine, Musgrove Park Hospital, Taunton, GBR

**Keywords:** gv bleeding, gastroesophageal varices, liver cirrhosis, evl, ppi

## Abstract

Background

Endoscopic variceal ligation (EVL) is a surgical intervention that can work well to curb variceal bleeding in people with liver cirrhosis. However, it could make ulcer bleeding worse and be fatal in some cases. The widespread use of proton pump inhibitors (PPI) in cirrhotic individuals with variceal bleeding is empirical rather than based on scientific data. According to many studies, PPIs reduce the size of post-EVL ulcers. This study aimed to see if PPI use could reduce rebleeding after endoscopy therapy in cirrhotic patients with variceal bleeding.

Methodology

A retrospective cross-sectional study was conducted at a tertiary care hospital from August 2019 to September 2021. Cirrhotic patients with bleeding gastroesophageal varices* *(GEVs) who had undergone EVL at the same hospital were enrolled in the study. Medical records were organized, and the sample was divided into two groups based on whether or not PPI was given. Both PPI and non-PPI patients had their endoscopic findings, initial hemostasis outcomes, rebleeding rates, bleeding-related mortality rates, and treatment-related comorbidities compared.

Results

A total of 46 patients were selected for the study and divided into two groups (PPI group n=28 and non-PPI group n=18). The majority of the patients were males. The PPI group had a mean age of 58.6 ±7.8 years, whereas the non-PPI group had a mean age of 53.6 ±4.4 years. Hepatitis B virus (HBV) infection was the most prevalent cause of cirrhosis in both groups. After endoscopic treatment, three patients (16%) in the non-PPI group suffered a variceal hemorrhage. Bleeding-related fatalities and the time it took for the bleeding to stop varied significantly between the two groups. History of variceal bleeding (relative risk (RR)=1.45; 95% confidence interval (CI), 1.60-7.67; p=0.02), presence of gastric varices (RR=2.23; 95% CI, 2.56-9.832; p=0.035), and not administering PPIs (RR =7.542; 95% CI, 3.98-29.13; p=0.008) were linked with rebleeding. The presence of red concurrent esophageal varices (RR=6.37; 95% CI, 0.562-15.342; p=0.002) and failure to provide PPIs (RR=2.3; 95% CI, 1.621-25.64; p=0.04) were linked with post-EVL bleeding in a multivariate analysis.

Conclusions

Proton pump inhibitors reduce the occurrence of early bleeding and adverse events after EVL in cirrhotic patients. Not prescribing PPIs and the presence of GEVs were substantially related to a higher risk of bleeding during preventative EVL. Not initiating PPI medication immediately was the sole predictor of bleeding complications in patients who had undergone EVL without gastric varix treatment. To lower the risk of post-EVL ulcer bleeding, we recommend PPI use in patients undergoing EVL.

## Introduction

Gastroesophageal varices (GEVs) are among the most prevalent complications in liver cirrhosis patients [1]. These are the enlarged blood vessels caused by portal hypertension. The incidence of GEVs amongst liver cirrhosis patients is reportedly 50% at the time of diagnosis [2]. The yearly incidence of first bleeding is 15%, with a mortality rate between 7% and 15%. Gastroesophageal varices are the most common cause of death in people with end-stage liver disease [3-7]. Many studies have proven the effectiveness of preventative approaches, including endoscopic and pharmaceutical therapy [8].

Few treatment strategies have proven to lower the occurrence of adverse bleeding events in GEVs. The most common approaches encompass the following: (1) endoscopic variceal ligation (EVL) or endoscopic injection sclerotherapy (EIS), and (2) prophylaxis with elective EVL or EIS and pharmacological treatment [9]. The search for effective, safe, and noninvasive treatments goes on, and proton pump inhibitors (PPIs) have been seen as a good option because they are safe for short-term use and work well for gastrointestinal (GI) bleeding that is not caused by varices [1]. Guidelines briefly address the use of PPIs in GEV to enhance the safety and effectiveness post-EVL [2,10].

Endoscopic variceal band ligation has been used to stop variceal bleeding, making early variceal bleeding much less common [11]. It is critical to note that between 2% and 5% of patients with EVL have fatal post-ligation ulcer hemorrhage. Perforation, substernal pain, transient dysphagia, stenosis, ulcer bleeding, and infection are additional risks of EVL. After the implantation of the bands on the varices, the ligated tissue may rupture. Superficial esophageal ulcers often develop at ligated sites of esophageal varices after the varices have molted [12,13].

In most cases, ligated-site ulcers are minor and resolve independently within a month. Bleeding induced by the band dislodging and exposing unhealed sores on the varices could be fatal, necessitating indefinite endoscopic surveillance [12,13]. Research shows that on follow-up endoscopies, patients who got a PPI after elective EVL had considerably fewer post-banding ulcers than those who received a placebo [14,15]. Several studies have examined whether PPIs could lower the risk of bleeding after EVL. Bleeding variants are more likely associated with higher portal pressure and decreased liver function, particularly in people who have previously experienced bleeding [16,17]. It has been shown that taking PPIs before EVL makes it less likely that the varices will come loose early, reduces the size of the ulcer by stopping acid production, and speeds up the healing process [18,19]. In addition, specific investigations have shown that PPI is ineffective [20,21]. Whether the PPI can reduce mortality is likewise unknown. Not much is written about how well PPIs work to stop bleeding during EVL. The current study will assist clinicians in making evidence-based judgments on PPI treatment in liver cirrhosis patients by filling a research gap. This study aimed to determine how well PPI works to stop upper GI bleeding in people with liver cirrhosis after EVL.

## Materials and methods

Subjects

In the current retrospective cross-sectional study at Ayub Teaching Hospital, Abbottabad, Pakistan, 46 patients with liver cirrhosis and GEVs were treated with EVL as a preventive intervention for variceal bleeding. Different doctors arbitrarily decided the prescription dose and duration of PPI. Patients with a history of acid reflux disease, intrahepatic thrombus, liver and esophageal surgery, hepatocellular malignancies, pregnancy or preparation to be pregnant, breastfeeding, or allergic reactions to PPIs were excluded from the study. The following information was gathered from medical records: (1) patient demographics; (2) serum biochemistry; (3) the cause of liver cirrhosis; (4) endoscopy findings; (5) deaths caused by bleeding (acute VH); (6) Child-Pugh scores; and (7) the rate and length of rebleeding. Post-EVL bleeding was defined as having bloody or bloody-tinged diarrhea within eight weeks of a preventive EVL. Also, post-EVL ulcer bleeding was identified if an endoscopy showed signs of GEVs. Follow-up endoscopies were performed before the patients were discharged from the hospital.

Endoscopic procedures for varices

A skilled endoscopist performed the EVL to avoid a first variceal bleed in patients with esophageal varices at high risk. If high-risk esophageal varices were found during the screening, the endoscope was removed, and an EVL device was implanted. After reinserting the endoscope, EVL was performed on the varix. After four to eight weeks, the ulcers had another endoscopy to make sure they were completely healed. A single endoscopist performed all procedures and operations using the same method outlined in prior investigations [22].

Ethical consideration

The ethics committee on human research of the Ayub Medical Institute examined and approved the study protocol (approval no. Re=325-AAA-ERC-MTI).

Statistical analysis

The SPSS version 26 (IBM Corp., Armonk, NY, USA) was used to conduct statistical analysis. The findings were shown as the mean and standard deviation (SD) for continuous variables and as a percentage (number) for categorical variables. The Pearson chi-square test and Fisher's exact test were conducted as applicable for analytical purposes. A p-value less than or equal to 0.05 was considered statistically significant.

## Results

Medical records of 89 patients were retrieved from the hospital. After exclusion, 46 individuals were enrolled and separated into two groups (based on PPI usage). The demographic and clinical features of the patients at baseline are shown in Table 1. The majority of patients in both groups were male. In both groups, the male-to-female ratio was 18:10 and 6:6, respectively. The PPI group had a mean age of 58.6 (SD 7.8) years, whereas the non-PPI group had a mean age of 53.6 (SD 4.4) years. The hepatitis B virus (HBV) infection was the most prevalent cause of cirrhosis in both groups. Both groups had similar levels of biochemical markers in their serum. In both groups, the majority of patients had Child-Pugh B grades. As demonstrated in Table 1, concomitant esophageal varices were found in 50% of the PPI group and 44% of the non-PPI group.

**Table 1:** Baseline clinical and demographic characteristics of study groups HBV: Hepatitis B virus, HCV: Hepatitis C virus, MELD: Model for end-stage liver disease, SD: Standard deviation, PPI: Proton pump inhibitor

Characteristics	PPI group (n=28)	Non-PPI group (n=18)
Age (mean±SD)	58.6 ± 7.8	53.6 ± 4.4
Gender (male:female)	18:10	12:6
Previous history of visceral bleeding n (%)	9 (32%)	7 (39%)
Etiology of cirrhosis n (%)		
HBV	21 (75%)	15 (83%)
HCV	0 (%)	0 (0%)
Autoimmune	3 (10.7%)	2 (11%)
Others	4 (14%)	1 (6%)
MELD score (mean±SD)	11±0.21	12±3.4
Child-Pugh degree n (%)		
Child-Pugh A	7 (25%)	3 (16.6%)
Child-Pugh B	16 (57%)	14 (77.7%)
Child-Pugh C	5(18%)	1 (5.7%)
Hemoglobin(g/L)	10.08± 4.18	11.41 ± 2.83
Platelets, 10^3^ cells/mL	99.38 ± 21.7	93.22 ± 38.02
Albumin, g/dL	2.32 ± 0.41	2.82 ±0.55
Prothrombin activity (mean±SD)	74 ± 9	70 ± 11
Total bilirubin, mg/dL	3.11 ± 0.5	5.52 ±1.30
Gastric varices n (%)		
F1	8 (28.5%)	3 (16.6%)
F2	6 (21.4%)	7 (38.8%)
F3	14 (50.1%)	8 (44.6%)
Concomitant esophageal varices n( %)		
F3 Red color (+)	14 (50%), 4 (14.1%)	8 (44.6%), 2 (11.1%)
Previous history of visceral bleeding n (%)	6 (21.4%)	8 (44.4%)
Follow-up duration, day (mean±SD)	202±35	185±21

When looking at bleeding episodes during the first two days after endoscopic treatment, neither group was at risk. Three patients in the non-PPI group (16%) had a variceal hemorrhage after endoscopic therapy. There was a notable distinction between the duration required for the bleeding to cease and the number of deaths caused by the bleeding. One patient with substantial variceal bleeding in the non-PPI group died of rebleeding after endoscopic treatment, even though five patients with bleeding episodes were successfully treated with medicines and endoscopic procedures (p=0.04). Adverse events at follow-up endoscopy were more in the non-PPI group than in the other group, but no statistical significance was observed (Table 2).

**Table 2:** Treatment outcomes and clinical characteristics at follow-up study * significant result EDG: Endoscopy, EVL: Endoscopic variceal ligation

Outcomes	PPI group (n=28)	Non-PPI group (n=18)	p-value
Early bleeding (within 48 hours) n (%)	0 (0%)	0 (0%)	0.68
Total bleeding (within 8 weeks) n (%)	2 (7%)	3 (16%)	0.05
Follow up EDG n (%)	6 (21%)	4 (22%)	0.54
Previous history of visceral bleeding n (%)	6 (21.4%)	8 (44.4%)	0.024*
Rebleeding interval (months) (mean ±SD)	10±6.5	12±6	0.03*
Bleeding-related deaths n (%)	0 (0%)	1 (5.5%)	0.04*
Blood transfusion (units) n (%)	0 (0%)	0 (0%)	1
Follow-up duration (days) (mean ±SD)	250.55±23.1	286±15.4	0.09
Child-Pugh score (mean ±SD)	7.4±1.5	9.3±2.2	0.07
Total adverse events EVL-induced ulcers at follow-up endoscopy n (%)	5 (17.8%)	8 (44%)	0.48

Factors affecting post-EVL bleeding: clinical and endoscopic perspectives

Clinical variables independently linked with post-EVL bleeding were hypoalbuminemia (RR=0.341; 95% CI, 0.066-0.892; p=0.013), Child-Pugh degree C (RR=5.102; 95% CI, 1.280-15.47; p=0.002), and HBV as the etiology (RR=2.36; 95% CI, 0.34-0.762; p=0.018).

The presence of gastric varices (RR=2.23; 95% CI, 2.56-9.832; p=0.035), previous history of visceral bleeding (RR=1.45; 95% CI, 1.60-7.67; p=0.025), and the non-usage of PPI medication (RR 7=.542; 95% CI, 3.98-29.13; p=0.008) were endoscopic and therapeutic variables independently related with bleeding after EVL (Table 3). Coexisting esophageal varices (RR=6.37; 95% CI, 0.562-15.342;p=0.002) and failure to provide PPIs (RR=2.3; 95% CI, 1.621-25.64; p=0.04) were statistically linked with EVL post-bleeding in a multivariate analysis.

**Table 3:** Univariate analysis for risk factors of bleeding * significant result RR: Relative risk, CI: Confidence interval, PPI: Proton pump inhibitor, HBV: Hepatitis B virus

Variable	RR	(95% CI)	p-value
Low albumin levels	0.341	0.066-0.892	0.012*
Child-Pugh degree C	5.102	1.280-15.47	0.002*
HBV	2.36	0.34-0.762	0.0018*
Gastric varices	2.23	2.56-9.832	0.035*
Previous history of visceral bleeding	1.45	1.60-7.67	0.025*
No PPI	7.542	3.98-29.13P	0.08*

## Discussion

Endoscopic variceal band ligation is advised to avoid initial variceal bleeding episodes in individuals with liver cirrhosis. The surgery is thought to be more successful than b-blockers, although this is still up for discussion [8,23]. However, complications such as bleeding from band-induced ulcerations have been linked to the surgery and its aftermath [2,13]. Although there is a lack of evidence, it is hypothesized that reducing stomach acid by PPI after EVL may help minimize postprocedural bleeding by decreasing the development of gastric ulcers.

For the prevention of GEVs bleeding, EVL treatment has primarily replaced injectable sclerotherapy since it is simpler to do, has a quicker therapeutic effect, and has fewer risks [10]. For this reason, it is essential to define the function of PPIs in conjunction with esophageal valproate therapy. Patients treated with pantoprazole following therapeutic endoscopy had more minor ulcers after endoscopic therapies than those treated with a placebo, according to a randomized study that included 42 subjects [15]. However, the research showed no connection between PPI usage and the danger of bleeding following preventive EVL. Recent research found that patients who used PPIs long-term had a lower chance of therapeutic failure after EVL [18]. The PPI therapy's effects on the active state of post-banding ulcerations were not assessed since all patients in the trial were randomly assigned after confirmation of post-EVL ulcer resolution.

In the current research, we assessed the impact of PPIs on post-EVL ulcer bleeding in 46 patients. Patients who received PPI medication had a considerably higher percentage of bleeding-free survival eight weeks after EVL. According to multivariate analysis, the absence of PPI administration and the presence of stomach varices were endoscopic and treatment-independent risk factors for bleeding following endoscopic therapy. By decreasing gastric acid reflux in cirrhotic patients, acid suppression is believed to have helped the rapid healing of post-banding ulcers. There is a possibility that patients who had combined endoscopic treatment for gastric varix might have a higher risk of bleeding after EVL. We hypothesize that starting proton pump inhibitor medication as soon as feasible after EVL is essential for preventing postprocedural hemorrhage.

Research has shown that the degree of liver damage is a significant predictor of unfavorable events following endoscopy and variceal rebleeding within eight weeks; Child-Pugh class C has been linked to bleeding after end-stage liver cirrhosis [17,24,25]. Using univariate analysis, the current study found a correlation between a Child-Pugh C degree and an increased risk of bleeding after primary EVL. However, this effect was not sustained using multivariate analysis, which accounted for potential confounders. Factors such as liver function, coagulopathy, portal pressure, and the size of varices (with or without bleeding stigmata) are more critical in cirrhotic patients who have experienced a variceal bleeding episode in the past. However, in patients who have never experienced variceal bleeding, the most crucial factor appears to be related to postprocedural management, such as the administration of PPIs.

Included among the study's strengths is the fact that the patient group was homogeneous; every patient in this research was treated with a single approach according to departmental standard operating procedures (SOPs) by the same physicians. Second is the fact that we were able to evaluate the long-term effects after EVL. As this research was retrospective and focused on the occurrence and control of post-banding ulcer bleeding after EVL, we had a longer than eight-week follow-up.

This research has some limitations. Firstly, the small sample size. This is because in clinical practice, we typically do not record every patient's detailed demographics and thus a detailed history was available for only a certain number of patients. Secondly, a variety of PPI medicines were provided which could present potential bias to the current finding. Future research might benefit more from comparing one or two types of PPI against a placebo. However, our data might still be utilized to interpret the findings, given there is no evidence to suggest that the impact of PPI type on post-EVL ulcer healing is distinct.

## Conclusions

There was a strong link between not giving PPIs and having stomach varices and an increased risk of bleeding after preventive EVL. Not beginning PPI medication was the sole predictor of bleeding complications in patients who got EVL without gastric varix treatment. Patients getting EVL may consider PPIs to minimize the risk of post-EVL ulcer bleeding.
